# Quality of life and patient satisfaction with raloxifene/cholecalciferol combination therapy in postmenopausal women

**DOI:** 10.1038/s41598-022-11298-2

**Published:** 2022-05-03

**Authors:** Dong-Yun Lee, Yoon-Sok Chung

**Affiliations:** 1 Department of Obstetrics and Gynecology, Samsung Medical Center, Sungkyunkwan University School of Medicine, Seoul, South Korea; 2 Department of Endocrinology and Metabolism, Ajou University School of Medicine, 164 World Cup-ro, Suwon, 16499 South Korea

**Keywords:** Diseases, Endocrinology

## Abstract

This study was performed to evaluate quality of life (QOL) and patient satisfaction with raloxifene/cholecalciferol combination therapy in postmenopausal women with low bone mass. This multicenter, prospective, noninterventional observational study included 3907 postmenopausal women who received a combination of raloxifene 60 mg and cholecalciferol 800 IU daily to treat or prevent osteoporosis. Changes in QOL and patient satisfaction were evaluated after 3 and 6 months of treatment. In addition, the safety profile was assessed. Mean age was 67.7 ± 9.3 years old. QOL, assessed by European Quality of life instrument 5 Dimensions (EQ-5D) index, improved significantly after 3 months (0.81 ± 0.11, *P* < 0.001) and 6 months (0.82 ± 0.11, *P* < 0.001) of treatment compared to the baseline (0.78 ± 0.14). Improvement in QOL was also significant regardless of previous regimens both in women who were switched from other drugs (bisphosphonates or selective estrogen receptor modulators) and in women who received the study drug for the first time (*P* < 0.001 for all comparisons). Percentage of women satisfied with the effects (from 37.3 to 67.7%, *P* < 0.001) and convenience (from 42.8 to 74.1%, *P* < 0.001) of treatment compared to previous medication significantly increased after 6 months of treatment. In addition, serious adverse drug reactions did not occur, and hot flushes were observed only in 12 women (0.3%). Combination therapy with raloxifene and cholecalciferol significantly improves quality of life with no serious adverse events and high patient satisfaction at 6 months. Our real-world data suggest that this regimen is a promising option for postmenopausal women with low bone mass.

## Introduction

Osteoporosis is an important health concern both medically and socioeconomically, since fractures due to osteoporosis are associated with high mortality and morbidity in older populations. Moreover, as life expectancy increases with time, the prevalence of osteoporosis is also increasing. The overall prevalence of osteoporosis is 34.8% in Korean postmenopausal women aged over 50^1^.

Many antiosteoporotic medications have been used in postmenopausal women with low bone mass. Among them, raloxifene, a selective estrogen receptor modulator (SERM), has proven efficacy for the prevention and treatment of osteoporosis, improving bone mineral density and reducing fracture risk^2,3^. In addition, it also demonstrates nonskeletal benefits, such as reduced breast cancer risk and a favorable safety profile^3,4^. Considering its efficacy and safety, raloxifene is widely used in postmenopausal women.

Vitamin D plays an important role in skeletal health. Although vitamin D alone may not be sufficient to reduce fracture risk, it could reduce fractures when combined with calcium, especially at a dose of ≥ 800 IU per day^5,6^. Meanwhile, vitamin D may also contribute to reducing falls^7^. Therefore, vitamin D is an essential adjunct to anti-osteoporotic medication, and maintaining adequate serum vitamin D is recommended by several osteoporosis treatment guidelines^8,9^. Nevertheless, the prevalence of vitamin D deficiency is high in Korea, mainly because of insufficient vitamin D-fortified food intake and sunlight exposure^10^.

Thus, a combination treatment containing SERM and vitamin D is expected to improve compliance and convenience as well as the effectiveness of osteoporosis treatment. In addition, beyond bone density and fracture, improvement in quality of life (QOL) is an important outcome in the treatment of osteoporosis^11^. Several studies have investigated the effects of anti-osteoporotic medications such as raloxifene or vitamin D alone on QOL^12–14^, but the effects of SERM and vitamin D combination therapy on QOL have not been fully evaluated^11^.

This prospective observational study was performed to evaluate QOL changes and patient satisfaction related to 6-month raloxifene/cholecalciferol combination therapy in postmenopausal women with low bone mass.

## Materials and methods

### Study population

This 6-month, multicenter, prospective, noninterventional observational study included postmenopausal Korean women with osteopenia or osteoporosis which were diagnosed based on World Health Organization criteria of bone mineral density at the lumbar spine and proximal femur measured by dual-energy X-ray absorptiometry.

Subjects were considered eligible for the study if they were postmenopausal women with osteopenia or osteoporosis who needed antiosteoporotic medication to prevent or treat osteoporosis. Women who had any contraindication to treatment were excluded from the study.

The study protocol was approved by the institutional review board of Samsung Medical Center and was conducted in accordance with the Declaration of Helsinki and local laws and regulations. Written informed consent was obtained from all eligible participants.

### Study design

The study period was from November 2017 to July 2020. Since this was an observational (noninterventional) study, start of medication at the clinician’s discretion. Clinicians enrolled the patient after explaining the study when a patient who met the inclusion/exclusion criteria was identified while conducting routine clinical practice.

At baseline (visit 1), informed consent was obtained from the study participant after providing detailed information about the study. Meanwhile, demographic (age, date of birth, height, and weight), smoking, and alcohol consumption data, as well as medical history (especially any treatment history of osteoporosis) were also obtained. QOL and satisfaction with previous medications were assessed using a questionnaire. In addition, laboratory tests, such as bone turnover markers and vitamin D, were newly measured or obtained from medical records if available. Study participants were asked to take one capsule of the study drug, Rabone D (Hanmi Pharma, South Korea), per day during the 6-month study period. Each Rabone D capsule contained raloxifene 60 mg and cholecalciferol 800 IU.

After 3 months (visit 2) and 6 months (visit 3) of treatment, QOL and satisfaction with Rabone D were assessed using the same questionnaire. Meanwhile, any adverse events were recorded. When laboratory tests were performed, results were also recorded.

### Assessment

#### Primary endpoint

QOL was evaluated using Korean version of European Quality of Life Instrument 5 Dimensions (EQ-5D) at each visit. EQ-5D, a healthcare survey, is composed of five dimensions such as mobility, self-care, usual activities, pain/discomfort, and anxiety/depression. In each dimension, which was rated on a 5-point scale corresponding to “no problems” (point 1), “slight problems” (point 2), “moderate problems” (point 3), “severe problems” (point 4), and “extreme problems” (point 5), a high score means unhealthy status. In order to examine the effects of Rabone D on EQ-5D and factors contributing to EQ-5D, we calculated the EQ-5D index by applying a formula that attaches weights to each of the scales in each dimension and subtracts its value from 1. Therefore, the higher index score indicates the higher health utility, by contrast with the score of individual questions. The weights for EQ-5D index were derived using time trade-off and discrete choice experiments based on a previous study^15^.

#### Secondary endpoint

Subjective patient satisfaction from the effects and convenience of treatment was assessed using a 5-point Likert scale questionnaire (very satisfied, satisfied, neither satisfied nor dissatisfied, dissatisfied, and very dissatisfied). Responses of “very satisfied” and “satisfied” were combined into “satisfied”, and those of “dissatisfied” and “very dissatisfied” were combined into “not satisfied” for analyses.

Changes in laboratory tests were also assessed, if available. However, since laboratory tests were not mandatory, measurement was decided based on clinical need.

Any adverse events observed during the study period were addressed.

### Statistical analysis

Data were collected using electronic case records from 99 sites in Korea (Supplementary Table 1). Considering the value of EQ-5D in a previous study, the alpha error (0.05), beta error (0.2), significance probability of EQ-5D size (0.01), and dropout rate (15%), the final sample size was calculated as 3,977.

For analyses, we divided the study subjects into three groups based on the following criteria: ‘full-analysis set’ (inclusion of participants who already had received other anti-osteoporotic medication), ‘safety set’ (inclusion of participants who were asked about adverse events), and ‘per-protocol set’ (exclusion of participants who were dropped or lost to follow-up).

Changes in quality of life, assessed by the EQ-5D, were analyzed according to the EQ-5D index, dimensions of the EQ-5D, and previous anti-osteoporotic treatment. We calculated estimated mean and standard deviation and performed repeated measures analysis of variance (ANOVA) after tests for normality to compare data at baseline, 3 months, and 6 months. When a significant difference was found in repeated measures ANOVA, post hoc test (Bonferroni) was applied for comparing paired data (baseline vs. 3 months, 3 months vs. 6 months, and baseline vs. 6 months). In repeated measures ANOVA, interaction effect term between time and group was considered. Changes in serum vitamin D level after treatment was also evaluated using repeated measures ANOVA and Bonferroni test in the same manner as the EQ-5D.

To identify the factors influencing the EQ-5D index change at each time from baseline, linear regression was used to evaluate the correlation between variables and differences of the EQ-5D index after treatment. We calculated coefficients to determine the estimated effects of each independent variable on the difference of the EQ-5D index.

Changes in patient satisfaction from effect and convenience after treatment were analyzed using McNemar test for paired sample.

Statistical analyses were performed using R statistic software version 3.2.2. Missing values were omitted. All tests were two-tailed and were performed at a *P* = 0.05 level of significance.

### Ethics approval

All procedures performed in studies involving human participants were in accordance with the ethical standards of the institutional and/or national research committee and with the 1964 Helsinki declaration and its later amendments or comparable ethical standards.

### Informed consent

Informed consent was obtained from all participants.

## Results

### Study participants

**Figure 1:**
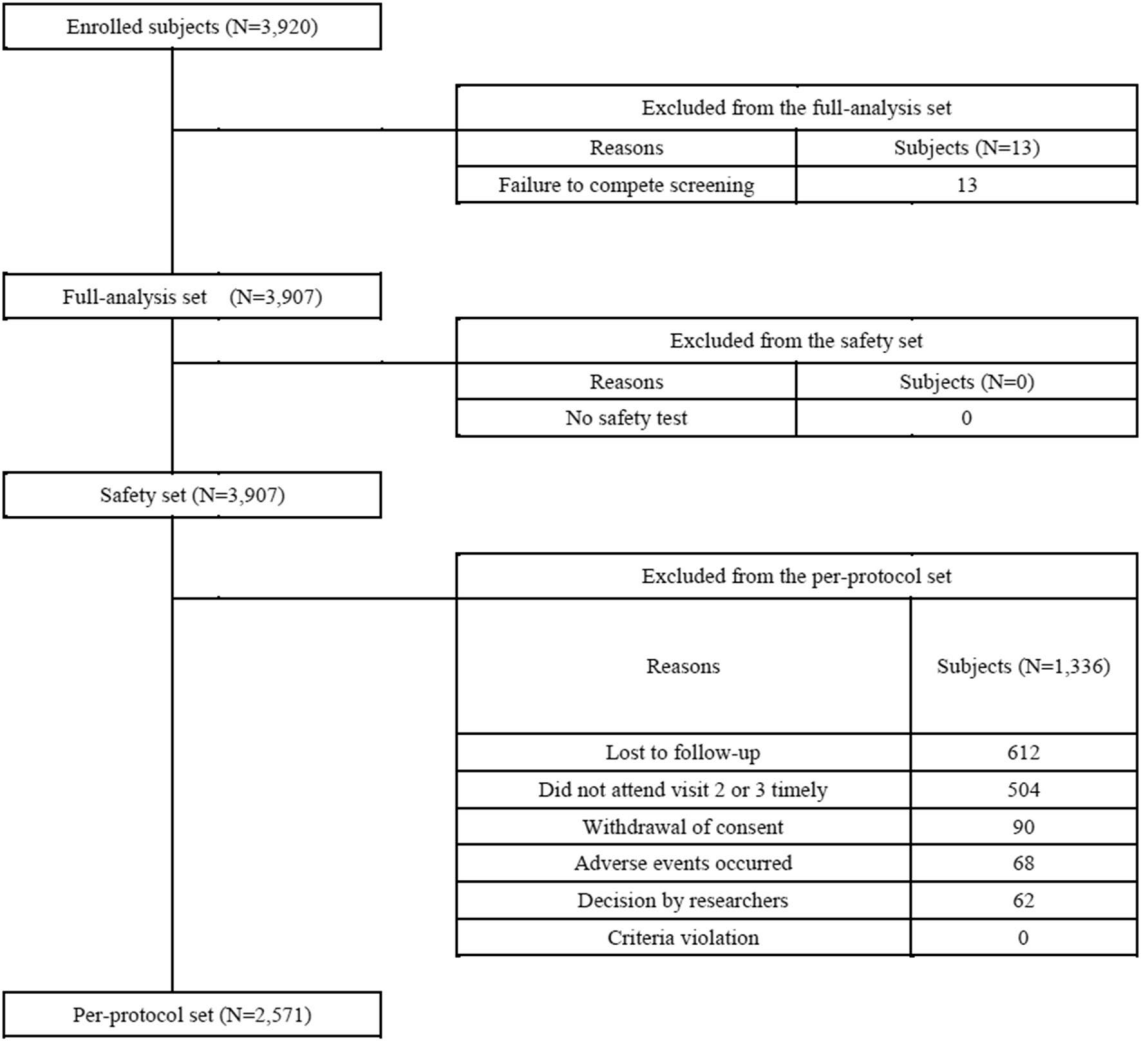
Flow chart of study participants.

Figure 1 depicts the flow chart of the study participants. Among 3,920 participants, 3,907 were included in the full-analysis set and safety set. During the study period, over 1,000 participants were excluded. Lost to follow-up (n = 612), and failure to attend follow-up visits in a timely manner (beyond 1 month, n = 504) were common reasons for exclusion. Finally, 2,571 participants were included in the per-protocol set.

Table 1 presents the baseline characteristics of the study participants. Mean age and body mass index were 67.7 ± 9.3 years old and 23.4 ± 3.1 kg/m^2^, respectively. The proportion of women who had a history of vertebral or nonvertebral fracture at baseline was 1.51% and 5.63%, respectively. In addition, 70.1% were diagnosed with osteoporosis, and 43.3% already took medications for the prevention or treatment of osteoporosis.

**Table 1:** Baseline characteristics of the study participants.

Characteristics	N = 3,907
Age (years)	67.7 ± 9.3
Height (cm)	153.9 ± 5.7
Weight (kg)	55.4 ± 7.8
Body mass index (kg/m^2^)	23.4 ± 3.1
**Current smoking**	
Yes	31 (0.79)
No	3,355 (85.87)
Unknown	521 (13.34)
**Alcohol use**	
Yes	140 (3.58)
No	3,214 (82.26)
Unknown	553 (14.15)
**History of fracture**^a,b^	
Vertebral fracture	59 (1.51)
Non-vertebral fracture	220 (5.63)
Diagnosed with osteoporosis	2,740 (70.13)
Prior treatment for osteoporosis	1,697 (43.43)

Data are presented as mean ± SD or number (percentage).

^a^SOC (System Organ Class), coding by Medical Dictionary for Regulatory Activities (MedDRA ver. 20.1).

^b^Overlapping count.

### Quality of life assessed by EQ-5D

Table 2 shows the change in EQ-5D during the study period. EQ-5D index had significantly improved after 3 months (0.81 ± 0.11) and 6 months (0.82 ± 0.11) of treatment compared to baseline (0.78 ± 0.14) (*P* < 0.001). Improvement was observed in all dimensions of EQ-5D.

**Table 2:** Changes in quality of life assessed by EQ-5D after treatment.

	Baseline	3 months	6 months	*P*-value^a^
EQ-5D index	0.78 ± 0.14	0.81 ± 0.11	0.82 ± 0.11	< 0.001^b^
**Dimensions**				
Mobility	1.76 ± 0.92	1.62 ± 0.82	1.57 ± 0.78	< 0.001^b^ for all dimensions
Self-care	1.43 ± 0.77	1.34 ± 0.66	1.30 ± 0.63	
Usual activities	1.67 ± 0.88	1.56 ± 0.78	1.49 ± 0.72	
Pain/discomfort	2.01 ± 0.87	1.81 ± 0.79	1.73 ± 0.74	
Anxiety/depression	1.54 ± 0.72	1.40 ± 0.61	1.36 ± 0.58	

Data are presented as mean ± SD.

^a^By repeated measures analysis of variance.

^b^Post hoc result by Bonferroni, there is a difference between all times (*P*-value < 0.001).

EQ-5D, European Quality of Life Instrument 5 Dimensions.

In addition, as a result of analyzing factors related to the EQ-5D index change after treatment, the higher age at baseline (by a 10-year grouping), the higher the EQ-5D index change is after both 3 and 6 months of treatment. Patient satisfaction from the effects also showed a significant positive correlation with a high EQ-5D index change, whereas satisfaction from convenience and the other factors did not have a significant association with the EQ-5D index change (Supplementary Table 2).

Table 3 demonstrates changes in EQ-5D index in women who were switched from other anti-osteoporotic medications to Rabone D and in women who received Rabone D as their first medication. Improvement in EQ-5D index was significant at each time point according to prior anti-osteoporotic medication (Group: *P* = 0.004, Time: *P* < 0.001). No differences were found in EQ-5D index across medications at 3 and 6 months of treatment.

**Table 3:** Changes in quality of life assessed by EQ-5D index according to previous anti-osteoporotic treatment.

Previous medication	Baseline	3 months	6 months	*P*-value^a^
No prior medications	0.79 ± 0.13	0.81 ± 0.11	0.82 ± 0.10	Group: 0.192 Time: < 0.001^c^ Group × time: 0.038
Prior medications	0.77 ± 0.14	0.81 ± 0.12	0.81 ± 0.12	
*P*-value ^a^	0.044	0.561	0.434	
Bisphosphonates	0.78 ± 0.14	0.80 ± 0.10	0.81 ± 0.11	Group: 0.004 Time: < 0.001^c^ Group × time: 0.570
Bisphosphonates, combined	0.79 ± 0.13	0.82 ± 0.09	0.83 ± 0.10	
SERMs	0.78 ± 0.14	0.80 ± 0.12	0.82 ± 0.10	
Other	0.79 ± 0.14	0.81 ± 0.13	0.83 ± 0.10	

Data are presented as mean ± SD.

*EQ-5D* European Quality of Life Instrument 5 Dimensions, *SERM* selective estrogen receptor modulator.

^a^By repeated measures analysis of variance.

^b^Calculated by independent t-test.

^c^Post hoc result by Bonferroni, there is a difference between all times (*P*-value < 0.001).

### Satisfaction

**Figure 2:**
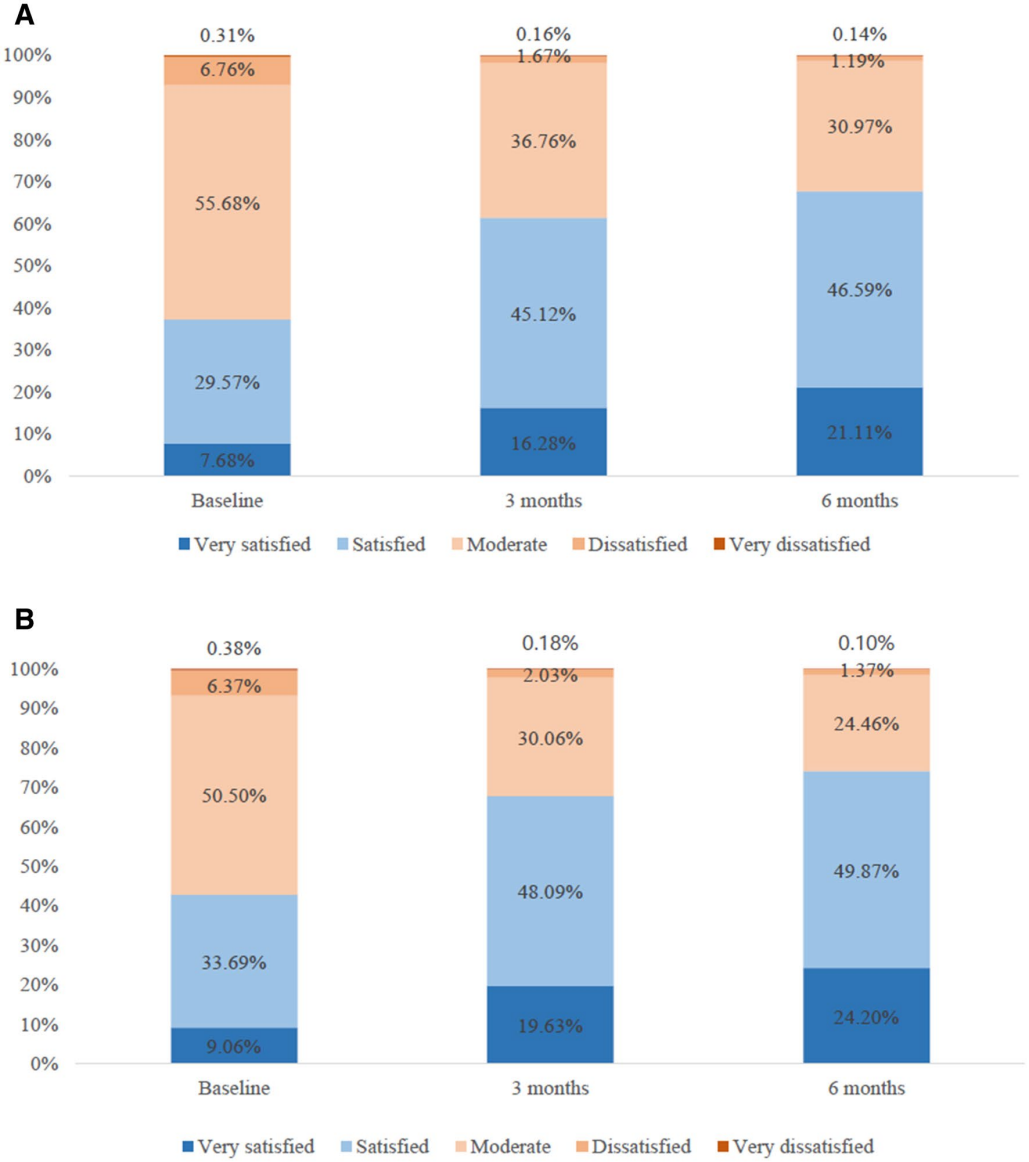
Changes in patient satisfaction from the effect (**A**) and convenience (**B**) of Rabone D use over the study period among patients previously taking other medications. For both satisfaction from effect and convenience, differences before and after treatment were statistically significant on McNemar test (*P* < 0.001 for all comparisons).

When evaluating patient satisfaction by questionnaire, percentage of women satisfied with both the effects (from 37.3 to 67.7%, *P* < 0.001) and convenience (from 42.8 to 74.1%, *P* < 0.001) of treatment significantly increased over 6 months in women who were switched from other anti-osteoporotic medication to Rabone D (Fig. 2).

### Adverse drug reactions

In addition, adverse drug reactions were reported using MeDRA (ver. 20.1) in 2.71%, and no one experienced a serious adverse drug reaction. Among adverse drug reactions, gastrointestinal disorders were the most common (n = 42). Hot flushes were observed in only 12 women (0.3%) (Table 4).

**Table 4:** Safety profile (n = 3,907).

	N (%)^a^	95% CI	Number of events
Treatment-emergent adverse event	323 (8.27)	[0.07, 0.09]	427
Unexpected adverse event	189 (4.84)	[0.04, 0.06]	231
Serious adverse event	28 (0.72)	[0.00, 0.01]	29
Adverse drug reaction^b^	106 (2.71)	[0.02, 0.03]	141
Unexpected adverse drug reaction	45 (1.15)	[0.008, 0.01]	55
Serious adverse drug reaction	0 (0.00)		0
**Adverse drug reaction**^c^			
Ear and labyrinth disorders	1 (0.03)		1
Eye disorders	1 (0.03)		1
Gastrointestinal disorders	42 (1.07)		45
General disorders and administration site conditions	16 (0.41)		16
Infections and infestations	2 (0.05)		2
Laboratory investigations	4 (0.10)		4
Metabolism and nutrition disorders	2 (0.05)		2
Musculoskeletal and connective tissue disorders	16 (0.41)		20
Nervous system disorders	18 (0.46)		18
Renal and urinary disorders	2 (0.05)		2
Reproductive system and breast disorders	4 (0.10)		4
Respiratory, thoracic and mediastinal disorders	3 (0.08)		4
Skin and subcutaneous tissue disorders	9 (0.23)		9
Vascular disorders	13 (0.33)		13
Hot flush	12 (0.30)		12
Hypertension	1 (0.03)		1

*CI* confidence interval.

^a^Overlapping count.

^b^Adverse drug reaction: certain, probable/likely, possible, conditional/unclassified, unassessable/unclassifiable.

^c^ Coding dictionary: MedDRA ver. 20.1.

### Serum vitamin D level

Table 5 presents changes in serum vitamin D level after treatment, if available. Serum vitamin D level significantly increased at 3 months of treatment compared to the baseline (*P* = 0.020 by Bonferroni), showing no significant difference between 3 and 6 months of treatment (*P* = 1.000 by Bonferroni).

**Table 5:** Changes in serum vitamin D level after treatment (n = 31).

	Baseline	3 months	6 months	*P*-value^a^
Serum vitamin D level (ng/mL)	23.90 ± 9.37	29.10 ± 7.82	28.90 ± 5.96	0.008

Data are presented as mean ± SD.

By post hoc analysis using Bonferroni, there was a significant difference between baseline and 3 months (*P* = 0.020). However, no difference was found between baseline and 6 months (*P* = 0.077) and between 3 and 6 months (*P* = 1.000).

^a^By repeated measures analysis of variance.

## Discussion

This prospective observational study evaluated QOL and patient satisfaction with effectiveness of raloxifene/cholecalciferol combination therapy in postmenopausal Korean women with low bone mass, and demonstrated that daily use of a raloxifene 60 mg and cholecalciferol 800 IU combination daily significantly improved QOL and patient satisfaction without serious adverse events.

Combination of anti-osteoporotic medication and vitamin D has been applied to enhance the effectiveness of and compliance with treatment for osteoporosis. Until now, combinations have mainly focused on bone health when vitamin D is added to bisphosphonates. In previous studies, a combination of a bisphosphonate, such as risedronate or ibandronate, and vitamin D produced higher serum vitamin D levels^9,16–19^ but did not increase bone mineral density (BMD) to a greater extent compared with bisphosphonate alone^16^. In addition, a decrease in serum PTH level was more pronounced in the combination group^16,17^. Vitamin D increases calcium absorption in the intestines and regulates bone turnover, and consequently prevents secondary hyperparathyroidism, which can lead to activation of osteoclasts and impaired bone mineralization^20^.

With regard to SERMs, a combination of raloxifene and vitamin D also produced a significantly lower PTH compared with raloxifene alone in a randomized study^21^. However, significant differences in BMD or bone turnover markers were not observed between the combination and raloxifene alone treatment groups at 12 or 24 months^21,22^. A combination of raloxifene and vitamin D has rarely been evaluated in terms of QOL. Raloxifene alone for 6 or 12 months was shown to improve EQ-5D in Japanese and European women^12,13^. Our results are consistent with a previous study demonstrating that 6-month combination therapy with raloxifene and alfacalcidol significantly improved QOL assessed using EQ-5D compared with baseline or raloxifene alone in 506 Japanese postmenopausal women with osteoporosis^11^.

In the current study, significant improvement in QOL was also observed in women who were switched from bisphosphonate or SERM alone. This finding suggests an additional benefit of vitamin D with raloxifene. Previous studies reported that a deficiency or low intake of vitamin D was associated with a significant decrease in physical or mental health-related QOL^14,23,24^. Thus, combination treatment can ensure a more stable and convenient supply of vitamin D.

In the present study, the proportion of women who experienced adverse events was very low, and the prevalence of hot flush, well-known adverse event related to SERM use, was less frequent than observed in Western countries^3,25^. This finding suggests that SERM is well tolerated in Asian postmenopausal women.

Although calcium is also important to bone health and adequate intake should be recommended with vitamin D, it is technically challenging to combine calcium with both a SERM and vitamin D in one capsule. In contrast to vitamin D, however, calcium can be obtained from food and routine supplementation is not recommended considering possibility of adverse effects.

The strength of the present study is in that it is a multicenter, prospective study in a large population of almost 4,000 postmenopausal women with low bone mass, reflecting real clinical practice. To further evidence-based medicine, randomized controlled clinical trials should have the highest priority. However, large-scale clinical trials are not always possible or feasible, due to limitations on time and resources. With a greater emphasis on effectiveness in specific medical environments, observational data is becoming more relevant, in spite of the inherent limitations of this study design. Data from patients in a real-world setting can better reflect routine clinical practice, improving our understanding of variable patterns of treatment, heterogeneous populations, alternative interventions, and variable patient monitoring^26,27^.

This study has several limitations. First, it was an observational study without a control (raloxifene only) group. Second, serum vitamin D levels were measured before and after treatment in only small number of subjects, since it was noninterventional observational study. However, as discussed earlier, a combination of antiosteoporotic medication and cholecalciferol was shown to produce significantly higher serum vitamin D levels in many other studies. In addition, due to the similar reasons, bone turnover markers were measured in a small proportion of the study subjects, and adequate statistical analysis was not possible. Third, the study duration (6 months) was relatively short, and was not adequate to assess BMD or fracture. However, the purpose of this study was not to investigate the efficacy of treatment on bone health, but to evaluate the effectiveness of treatment for QOL improvement and to determine patient satisfaction; a duration of 6 months was considered adequate for this purpose. Moreover, we had no detailed information regarding calcium intake, although we encouraged adequate calcium intake via food. And psychopathological history or cognitive impairment, which might be associated negatively with quality of life, were not assessed at the enrollment.

## Conclusion

Combination therapy of raloxifene and cholecalciferol significantly improved QOL without serious adverse events, leading to high patient satisfaction. Our real-world data suggest that this regimen is a promising option for postmenopausal women with low bone mass in real clinical practice.

## Supplementary Information

Supplementary Tables.

## References

[1] Choi MH, Yang JH, Seo JS (2021). Prevalence and diagnosis experience of osteoporosis in postmenopausal women over 50: Focusing on socioeconomic factors. PLoS ONE.

[2] Jolly EE, Bjarnason NH, Neven P (2003). Prevention of osteoporosis and uterine effects in postmenopausal women taking raloxifene for 5 years. Menopause.

[3] Ettinger B, Black DM, Mitlak BH (1999). Reduction of vertebral fracture risk in postmenopausal women with osteoporosis treated with raloxifene: Results from a 3-year randomized clinical trial. Multiple Outcomes of Raloxifene Evaluation (MORE) Investigators. JAMA.

[4] Martino S, Cauley JA, Barrett-Connor E (2004). Continuing outcomes relevant to Evista: Breast cancer incidence in postmenopausal osteoporotic women in a randomized trial of raloxifene. J. Natl. Cancer Inst..

[5] Avenell A, Mak JC, Oconnell D (2014). Vitamin D and vitamin D analogues for preventing fractures in post-menopausal women and older men. Cochrane Database Syst. Rev..

[6] Bischoff-Ferrari HA, Willett WC, Wong JB (2009). Prevention of nonvertebral fractures with oral vitamin D and dose dependency: A meta-analysis of randomized controlled trials. Arch. Intern. Med..

[7] Bischoff-Ferrari HA, Dawson-Hughes B, Staehelin HB (2009). Fall prevention with supplemental and active forms of vitamin D: A meta-analysis of randomised controlled trials. BMJ.

[8] Eastell R, Rosen CJ, Black DM (2019). Pharmacological management of osteoporosis in postmenopausal women: An endocrine society* clinical practice guideline. J. Clin. Endocrinol. Metab..

[9] Camacho PM, Petak SM, Binkley N (2020). American Association of Clinical Endocrinologists/American College of Endocrinology Clinical Practice Guidelines for the Diagnosis and Treatment of Postmenopausal Osteoporosis-2020 Update. Endocr. Pract..

[10] Choi HS, Oh HJ, Choi H (2011). Vitamin D insufficiency in Korea–a greater threat to younger generation: the Korea National Health and Nutrition Examination Survey (KNHANES) 2008. J. Clin. Endocrinol. Metab..

[11] Ohta H, Hamaya E, Taketsuna M (2015). Quality of life in Japanese women with postmenopausal osteoporosis treated with raloxifene and vitamin D: Post hoc analysis of a postmarketing study. Curr. Med. Res. Opin..

[12] Ringe JD, Christodoulakos GE, Mellström D (2007). Patient compliance with alendronate, risedronate and raloxifene for the treatment of osteoporosis in postmenopausal women. Curr. Med. Res. Opin.

[13] Yoh K, Hamaya E, Urushihara H (2012). Quality of life in raloxifene-treated Japanese women with postmenopausal osteoporosis: A prospective, postmarketing observational study. Curr. Med. Res. Opin..

[14] Ohta H, Uemura Y, Nakamura T (2014). Serum 25-hydroxyvitamin D level as an independent determinant of quality of life in osteoporosis with a high risk for fracture. Clin. Ther..

[15] Kim SH, Ahn J, Ock M (2016). The EQ-5D-5L valuation study in Korea. Qual. Life Res..

[16] Park SY, Kang MI, Park HM (2019). Efficacy of risedronate with cholecalciferol on bone mineral density in Korean patients with osteoporosis. Arch. Osteoporos..

[17] Chung HY, Koo J, Kwon SK (2013). Early changes in 25-hydroxyvitamin D levels and bone markers after monthly risedronate with cholecalciferol in Korean patients with osteoporosis. Clin. Interv. Aging.

[18] Chung HY, Chin SO, Kang MI (2011). Efficacy of risedronate with cholecalciferol on 25-hydroxyvitamin D level and bone turnover in Korean patients with osteoporosis. Clin. Endocrinol. (Oxf).

[19] Cho IJ, Chung HY, Kim SW (2015). Efficacy of a once-monthly pill containing ibandronate and cholecalciferol on the levels of 25-hydroxyvitamin D and bone markers in postmenopausal women with osteoporosis. Endocrinol. Metab. (Seoul).

[20] Thacher TD, Clarke BL (2011). Vitamin D insufficiency. Mayo Clin. Proc..

[21] Gorai I, Hattori S, Tanaka Y (2012). Alfacalcidol-supplemented raloxifene therapy has greater bone-sparing effect than raloxifene-alone therapy in postmenopausal Japanese women with osteoporosis or osteopenia. J. Bone Miner. Metab..

[22] Majima T, Komatsu Y, Shimatsu A (2008). Efficacy of combined treatment with raloxifene and alfacalcidol on bone density and biochemical markers of bone turnover in postmenopausal osteoporosis. Endocr. J..

[23] Basaran S, Guzel R, Coskun-Benlidayi I (2007). Vitamin D status: effects on quality of life in osteoporosis among Turkish women. Qual. Life Res..

[24] Motsinger S, Lazovich D, MacLehose RF (2012). Vitamin D intake and mental health-related quality of life in older women: The Iowa Women's Health Study. Maturitas.

[25] Silverman SL, Christiansen C, Genant HK (2008). Efficacy of bazedoxifene in reducing new vertebral fracture risk in postmenopausal women with osteoporosis: Results from a 3-year, randomized, placebo-, and active-controlled clinical trial. J. Bone Miner. Res..

[26] Heikinheimo O, Bitzer J, García Rodríguez L (2017). Real-world research and the role of observational data in the field of gynaecology–a practical review. Eur. J. Contracept Reprod. Health Care.

[27] Kim HS, Lee S, Kim JH (2018). Real-world evidence versus randomized controlled trial: Clinical research based on electronic medical records. J. Korean Med. Sci..

