# Supplementary material for: Quality of life and patient satisfaction with raloxifene/cholecalciferol combination therapy in postmenopausal women
Source: Sci Rep. 2022 May 3;12:7142. doi: 10.1038/s41598-022-11298-2 (PMC9065022; doi:10.1038/s41598-022-11298-2)
Supplement: Supplementary file 1 — Supplementary Tables. [file 41598_2022_11298_MOESM1_ESM.docx]

**Supplementary Table 1. List of participating institutions and principal investigators (PI)**

| **Institution Name** | **PI Name** |
| --- | --- |
| Hoyounkim's Clinic for Arthritis & Rheumatism | Ho-youn Kim |
| Sungmo OS Clinic | Sung-won Lim |
| Dr. Lee's Family Clinic | Myeong-chun Lee |
| Dr. Lee's Internal Medicine Clinic | Na-young Lee |
| Lee Internal Medicine Clinic | Sang-hyuk Lee |
| Ilsanmodeun Internal Medicine Clinic | Ki-deuk Nam |
| Jeonjunha Internal Medicine Clinic | Jun-ha Jeon |
| Hanjeongyeol Internal Medicine Clinic | Jeong-yeol Han |
| Guroseran Clinic | Hae-kyung Sa |
| Kimkyungho Internal Medicine Clinic | Kyung-ho Kim |
| Do's Neurologic Clinic | Byung-ryong Do |
| Dongyang Goodmorning Radiology Clinic & Healthcare Center | Jin-hee Lee |
| Dongyang Goodmorning Radiology Clinic & Healthcare Center | Jong-tae Jee |
| Rheuma Internal Medicine | Jae-ki Koh |
| Mokpocity Medical Center | Chol-hong Park |
| Park's Familiy Medicine Clinic | Keum-soo Park |
| Parkyongjoo Internal Medicine Clinic | Young-joo Park |
| Bokeum Internal Medicine Clinic | Chung-su Park |
| Samsung Clinic | Nak-cheon Kim |
| Saeseoul Medical Clinic | Min-kyeong Kim |
| Saeseoul Medical Clinic | Sang-im Jeon |
| Seoulsok Internal Medicine Clinic | Jeong-goo Kim |
| Seoulsok Internal Medicine Clinic | Wook-hyun Um |
| Somoonnan Internal Medicine Clinic | Byung-hoon Kim |
| Song Chang Hun Internal Medicine Clinic | Chang-hun Song |
| Yu-wunyong Medicine Clinic | Wun-yong Yu |
| Incheon Clinic | Kyu-jin Joo |
| Dr. Chang's Medical Center | Kyu-man Chang |
| Dr. Jo Medical Clinic | Jung-kon Cho |
| Hana Internal Medicine Clinic | Chan-woong Park |
| Hyosung Sarang Orthopedics Clinic | Won-seok Chae |
| Songtan-Jungang Hospital | Sung-won Yang |
| Hansarang Rehabitaion Clinic | Seong-yong Kim |
| Bokeum Internal Medicine Clinic | Gu-huck Jung |
| Modoo Family Care Center | Jae-pil Lee |
| Gachon University Gil Medical Center | Kwang-beom Lee |
| Chungnam National University Hospital | Yong-bum Joo |
| Jeju National University Hospital | Kwang-woo Nam |
| Cheju Halla General Hospital | Seong-hwan Kim |
| Hallym University Kangnam Sacred Heart Hospital | Kyu-chul Noh |
| Kyunghee University Hospital | Min-hyung Jung |
| Hanyang University Guri Hospital | Ye-soo Park |
| Hallym University Dongtan Sacred Heart Hospital | Il Choi |
| Hallym University Sacred Heart Hospital | Young-han Park |
| Seoul Metropolitan Government Seoul National University Boramae Medical Center | Soong-joon Lee |
| Seoul National University Bundang Hospital | Jung-ryeol Lee |
| Hanil General Hospital | Mi-seon Shin |
| Bundang Jesaeng Hospital | Yu-hun Jung |
| Chosun University Hospital | Yun-sung Kim |
| Kangdong Sacred Heart Hospital | Chong-soo Moon |
| Chonnam National University Hospital | Jung-kil Lee |
| Kyung Hee University Hospital | Kyung-chung Kang |
| Maryknoll Hospital | Ho-sang Kim |
| Soonchunhyang University Bucheon Hospital | Hae-hyeog Lee |
| Soonchunhyang University Bucheon Hospital | Byung-sung Kim |
| Konkuk University Medical Center | Ji-young Lee |
| Inje University Busan Paik Hospital | Dae-hyun Park |
| Severance Hospital | Yu-mie Rhee |
| Samsung Medical Center | Joon-ho Wang |
| Daegu Catholic Univ. Medical Center | Jung-yoon Choe |
| Inje University Haeundae Paik Hospital | Seong-su Kim |
| Dongguk University Medical Center | Sang-ho Yoon |
| Myohgji Hospital | Sang-heon Song |
| Myohgji Hospital | Jae-hyuk Lee |
| Dong-A University Hospital | Min-woo Kim |
| St. Carollo Hospital | Se-jin Kim |
| Cha University Bundang Medical Center | Tae-keun Ahn |
| Soon Chun Hyang University Cheonan Hospital | Sang-hye Jang |
| Seoul National University Bundang Hospital | Young-kyun Lee |
| Konkuk University Medical Center | Sang-heon Lee |
| Korea University Ansan Hospital | Se-hoon Kim |
| Soon Chun Hyang University Cheonan Hospital | Chang-hwa Hong |
| Soon Chun Hyang University Seoul Hospital | Jae-chul Lee |
| Samsung Medical Center | Dong-yun Lee |
| Hallym University Medical Center | Seok-woo Kim |
| Seoul Medical Center | Tae-ho Kim |
| Presbyterian Medical Center | Chun-shik Shin |
| Busan St. Mary’s Hospital | Chang-won Lee |
| Inje University Ilsan Paik Hospital | Bo-young Yoon |
| Kyung Hee University Hospital | Seung-jae Hong |
| Soonchunhyang University Bucheon Hospital | Ji-oh Mok |
| Bundang Jesaeng Hospital | Yong-hyun Kim |
| Korea University Guro Hospital | Jae-hoon Kim |
| Chung-Ang University Hospital | Kwang-sup Song |
| Ajou University Hospital | Yoon-sok Chung |
| Hallym University Chuncheon Sacred Heart Hospital | Yong-jun Cho |
| Chungbuk National University Hospital | Hyun-jeong Jeon |
| Chosun University Hospital | Chang-il Ju |
| Gangneung Asan Hospital | Sung-soo Kim |
| Inje University Sanggye Paik Hospital | Jung-yun Choi |
| Ajou University Hospital | Ye-yeon Won |
| Korea University Anam Hospital | Tak Kim |
| Ulsan University Hospital | Jae-ryong Cha |
| Soon Chun Hyang University Gumi Hospital | Dae-geun Kim |
| National Health Insurance Service Ilsan Hospital | Sang-hoon Park |
| Gwangju Veterans Hospital | Hong-man Cho |
| Yeungnam University Hospital | Chul-hyun Park |
| Seoul National University Hospital | Seok-hyun Kim |
| Konyang University Hospital | Youn-mu Heo |

**Supplementary Table 2. Factors influencing EQ-5D Index change***

| Variables | Difference between  3 months and baseline | | | Difference between  6 months and baseline | | |
| --- | --- | --- | --- | --- | --- | --- |
|  | β | SE | p-value | β | SE | p-value |
| Age | 0.00058 | 0.00021 | 0.0042 | 0.00122 | 0.00024 | <0.001 |
| Height | -0.00019 | 0.00036 | 0.5960 | -0.00031 | 0.00042 | 0.460 |
| Weight | 0.00650 | 0.00027 | 0.0151 | 0.00007 | 0.00030 | 0.825 |
| Body mass index | 0.00188 | 0.00067 | 0.0053 | 0.00041 | 0.00077 | 0.594 |
| Current smoking |  |  |  |  |  |  |
| Yes | 0.02155 | 0.02077 | 0.3000 | -0.00338 | 0.02422 | 0.889 |
| No | - | - | - | - | - | - |
| Unknown | -0.00060 | 0.00677 | 0.9300 | -0.00477 | 0.00757 | 0.529 |
| Alcohol use |  |  |  |  |  |  |
| Yes | 0.00440 | 0.00951 | 0.6430 | 0.00855 | 0.01085 | 0.431 |
| No | - | - | - | - | - | - |
| Unknown | -0.00212 | 0.00644 | 0.7420 | -0.00830 | 0.00724 | 0.252 |
| Diagnosed with osteoporosis |  |  |  |  |  |  |
| Yes | 0.00219 | 0.00410 | 0.5930 | 0.00101 | 0.00474 | 0.832 |
| No | - | - | - | - | - | - |
| Prior treatment for osteoporosis |  |  |  |  |  |  |
| Yes | 0.00175 | 0.00395 | 0.658 | 0.00192 | 0.00453 | 0.672 |
| No | - | - | - | - | - | - |
| Medication adherence |  |  |  |  |  |  |
| Adherence | 0.00618 | 0.00791 | 0.4346 | -0.00365 | 0.00851 | 0.668 |
| Non-adherence | - | - | - | - | - | - |
| Satisfaction from effects |  |  |  |  |  |  |
| Satisfied | 0.00861 | 0.00389 | 0.0269 | 0.01257 | 0.00457 | 0.006 |
| Not satisfied | - | - | - | - | - | - |
| Satisfaction from convenience |  |  |  |  |  |  |
| Satisfied | 0.00322 | 0.00394 | 0.414 | 0.00681 | 0.00484 | 0.159 |
| Not satisfied | - | - | - | - | - | - |
| ^*^Linear regression  SE, standard error |  |  |  |  |  |  |
